# Renal Contributions in the Pathophysiology and Neuropathological Substrates Shared by Chronic Kidney Disease and Alzheimer’s Disease

**DOI:** 10.3390/brainsci10080563

**Published:** 2020-08-17

**Authors:** Gabriela Dumitrita Stanciu, Daniela Carmen Ababei, Veronica Bild, Walther Bild, Luminita Paduraru, Mihai Marius Gutu, Bogdan-Ionel Tamba

**Affiliations:** 1 Center for Advanced Research and Development in Experimental Medicine (CEMEX), “Grigore T. Popa” University of Medicine and Pharmacy, 16 Universitatii street, 700115 Iasi, Romania; gabriela-dumitrita.s@umfiasi.ro (G.D.S.); bogdan.tamba@umfiasi.ro (B.-I.T.); 2 Pharmacodynamics and Clinical Pharmacy Department, “Grigore T. Popa” University of Medicine and Pharmacy, 16 Universitatii street, 700115 Iasi, Romania; veronica.bild@gmail.com; 3 Department of Physiology, “Grigore T. Popa” University of Medicine and Pharmacy, 16 Universitatii street, 700115 Iasi, Romania; walther.bild@umfiasi.ro; 4 Department Mother & Child Care, Division Neonatology, “Grigore T. Popa” University of Medicine and Pharmacy, 16 Universitatii street, 700115 Iasi, Romania; 5 Department of Biophysics and Medical Physics-Nuclear Medicine, “Grigore T. Popa” University of Medicine and Pharmacy, 16 Universitatii street, 700115 Iasi, Romania; marius.gutu@umfiasi.ro; 6 Department of Pharmacology, Clinical Pharmacology and Algesiology, “Grigore T. Popa” University of Medicine and Pharmacy, 16 Universitatii street, 700115 Iasi, Romania

**Keywords:** chronic kidney disease, Alzheimer’s disease, cognitive impairment, neuropathological substrates, pathophysiology

## Abstract

Chronic kidney disease and Alzheimer’s disease are chronic conditions highly prevalent in elderly communities and societies, and a diagnosis of them is devastating and life changing. Demanding therapies and changes, such as non-compliance, cognitive impairment, and non-cognitive anomalies, may lead to supplementary symptoms and subsequent worsening of well-being and quality of life, impacting the socio-economic status of both patient and family. In recent decades, additional hypotheses have attempted to clarify the connection between these two diseases, multifactorial in their nature, but even so, the mechanisms behind this link are still elusive. In this paper, we sought to highlight the current understanding of the mechanisms for cognitive decline in patients with these concurrent pathologies and provide insight into the relationship between markers related to these disease entities and whether the potential biomarkers for renal function may be used for the diagnosis of Alzheimer’s disease. Exploring detailed knowledge of etiologies, heterogeneity of risk factors, and neuropathological processes associated with these conditions opens opportunities for the development of new therapies and biomarkers to delay or slow their progression and validation of whether the setting of chronic kidney disease could be a potential determinant for cognitive damage in Alzheimer’s disease.

## 1. Prevalence, Socio-Economic Aspects, and the Relationship between Chronic Kidney Disease and Alzheimer’s Disease

Globally, dementia represents one of the most important social, economic, and public health challenges with extended human life expectancy. Epidemiological survey has estimated that ~50 million individuals around the world suffered from dementia in 2018, with Alzheimer’s disease (AD) comprising 60 to 80% of all cases, and the number is projected to triple by 2050 [1,2], mostly due to an elderly population but also to a growing prevalence of risk factors for dementia. The estimated total annual worldwide costs for care of AD people was USD 1 trillion in 2018, and this figure will rise to approximately USD 2 trillion by 2030. The fundamental risk factor for the development of AD is increased age [3,4,5]. Other recognized risk factors include family history [6], degeneration or vascular dysfunction [7,8], obesity [9], hypotension or hypertension [10], diabetes [11,12,13], hyperlipidemia [14,15], low levels of education, physical inactivity [16], and the existence of epsilon 4 allele of the apolipoprotein E gene (ApoE4) [6,17]. A recently proposed modifiable risk factor for AD is kidney disease. The amyloid precursor protein (APP) expression level in kidney disease patients is higher. It is a key protein for protein-bound receptor sorting (SorLA), which acts as a central regulator of APP trafficking and processing and is expressed concurrently in both neurons (cerebellum, hippocampus, and cortex), renal cells, and gene polymorphism, which is associated with late-onset AD [18,19,20,21,22,23]. 

Chronic kidney disease (CKD), also known as chronic kidney failure, defines a gradual loss of renal function that persists for three or more months; it is an essential contributor to mortality and morbidity from non-communicable conditions. The progression of disease is divided into a five distinct-stage system, as defined by the Kidney Disease Outcomes Quality Initiative (KDOQI) Clinical Practice Guidelines (Table 1) [24], focused on the estimated glomerular filtration rate (eGFR), a calculation of waste cleared by the kidneys per minute [25]. According to existing estimates, 697.5 million people (9.1%) were afflicted by CKD worldwide, with CKD stages 1–2 accounting for 5%, stage 3 for 3.9%, stage 4 for 0.16%, stage 5 for 0.07%, dialysis for 0.041%, and kidney transplantation for 0.011%, causing 1.2 million deaths in 2017 [26]. In recent decades, the costs of CKD therapy have increased with accessibility of renal replacement techniques [27], whereas over 2.5 million people have benefited from replacement therapy so far, and it is expected to double to 5.4 million by 2030 [28]. However, the most expensive treatment seems to be for patients with end-stage renal disease (ESRD). In the latest report, the Centers for Disease Control and Prevention announced costs of USD 84 billion for CKD of which USD 36 billion only was for ESRD [26]. 

**Figure 1:**
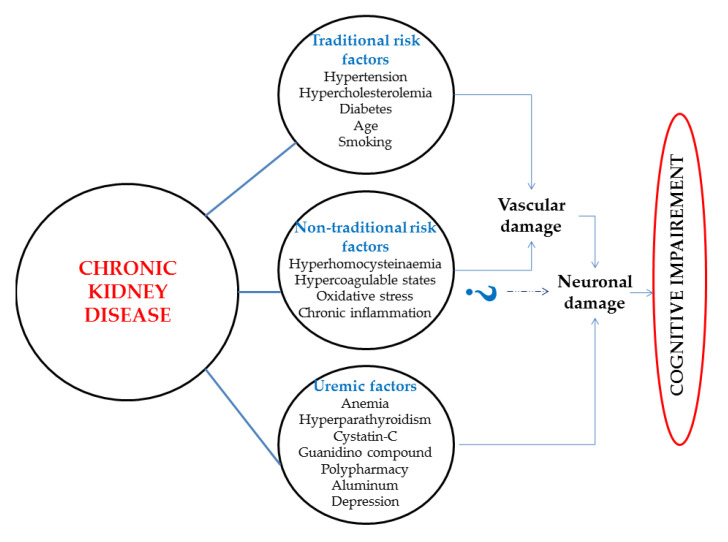
Factors linking chronic kidney disease and Alzheimer’s disease.

CKD can be initiated by a renal condition or can occur as a result of complications caused by multisystem disorders associated with comorbidities, such as diabetes mellitus type 2, which at present represents the most important factor of this disease globally. This condition is considered to be an independent risk factor for cognitive decline and dementia [25,29,30]. Dementia represents an essential complication, as it may lead to reduced medical adherence education and health literacy and is a robust, independent predictor of mortality in individuals suffering from dialysis [31]. Cognitive impairment prevalence in dialysis people has been described to be around 30 to 60% [32], and cases under hemodialysis have lower cognitive outcomes, mainly in attentional control, executive function fields, and orientation compared with patients on peritoneal dialysis [31,33]. The relationship between cognitive decline and CKD can be clarified by some factors, such as traditional risk factors, non-traditional risk factors, increased inflammation, and oxidative stress (Figure 1) [25,32,34]. Anemia, polypharmacy, hyperparathyroidism, depression, and sleep disorders may represent a supplementary link between cognitive decline and CKD. Moreover, patients under dialysis are exposed to hypoxemia, proinflammatory state, extensive fluid and osmolar shifts, and fluctuant concentrations of uremic toxins [32]. The latest data reveal that both the occurrence and evolution of cognitive decline are inversely related with the CKD stage. A current meta-analysis of cross-sectional and longitudinal studies including 54,779 people revealed that for every 10 mL reduction in the eGFR value below 60 mL/min/ 1.73 m^2^ an intensification is registered in the risk of cognitive impairment of 11% [35]. Furthermore, another part of the studies showed a more accelerated decrease in cognitive abilities over time when CKD is present [31,36]. 

CKD stimulates the development of cognitive decline and the progression of AD [25], which can prove to be an expediency but also a challenge in the early diagnosis and therapy of this chronic irreversible condition; additionally, the prevalence of CKD is constantly increasing. In the first multicenter study on CKD in China, Zhang et al. [8] reported an increase in AD of up to 10.8% in 50,550 CKD individuals, a rate that has shown an upward tendency year by year. 

To date, the pathophysiology of AD and the role of CKD in AD progression are not completely known. As effective pharmacotherapies of AD remain limited [37,38,39], studies based on the prevention against major, modifiable risk factors will matter more and more. In the current review, we sought to provide an overview of prevalence, socio-economic aspects, and links between CKD and AD. Furthermore, we highlight the present understanding of the mechanisms for cognitive decline in patients with these concurrent pathologies and provide insight into the relationship between markers related to AD and CKD and whether the potential biomarkers for renal function may be used for the diagnosis of Alzheimer’s disease. Detailed exploration of knowledge about the molecular link between these conditions opens new windows for diagnosis and treatment, the development of biomarkers, or the validation of whether the setting of CKD could be a possible new element for cognitive damage in AD. 

## 2. The Mechanisms Proposed for Cognitive Decline and Alzheimer’s Disease Associated with Chronic Kidney Disease

Despite the recognized association between cognitive conditions and renal failure, direct evidence connecting brain injury to CKD is still absent. In this context, various hypotheses have been designed as supplementary pathways for kidney–brain communication, comprising renin–angiotensin system, oxidative stress, vascular injury, and inflammation [40]. It is worth noting that the crosstalk between kidney and brain appears to be bidirectional, as conditions of the central nervous system, such as traumatic brain injury and migraine, are independent risk factors for CKD as well [41,42].

### 2.1. Vascular Dysfunction

From a pathophysiological point of view, it is known that AD may have a vascular constituent, and the cause of cognitive decline is multifaceted. In difference, individuals with CKD are expected to present a disproportionate degree of cerebrovascular disease (CVD), mostly small-vessel CVD, which might be a significant cause in the development and evolution of CKD-associated cognitive decline [43,44]. This theory is based on the fact that secondary neuropsychiatric disorders occurring in patients with renal lesions might be due to the hemodynamic relations between the kidney and the brain, CKD being acknowledged as an important cause for vascular dementia and stroke [45,46]. Vascular cognitive deficits or mixed vascular dysfunction and AD appear to have a much higher incidence in hemodialysis individuals than AD alone [47,48]. Therefore, there is a robust likelihood that CKD patients are at an elevated risk for cognitive decline determined by vascular-associated causes, expressed as brain microinfarcts and white matter disease, and not overt AD per se [49]. The cerebral vascular condition seems to act concurrently with a neurodegenerative mechanism partially facilitated by uremic toxins, homocysteine, cystatin C, and/or creatinine levels [50]. The cognitive damage reported in cerebrovascular conditions mostly influences processing speed, and executive functioning, and cognitive areas that influence planning and carry out a task, and the majority of findings have shown that executive function and processing are the domains most affected in CKD patients [51,52]. Finally, cognitive decline leads to worse emotional well-being and quality of life [53]. Reduction in renal function has been correlated with deficient cerebral white matter integrity, and the presence of albuminuria has been correlated with a diminution of glomerular filtration rate (GFR) and lower brain–blood flow [54]. As the renal function decreases in the patients with CKD, the cognitive performance worsens, but it may be improved by kidney transplant. Several longitudinal studies showed an improvement of cognitive function after transplantation, an effect explained by the fact that, following transplant, the essential functions of the kidneys may be restored on one side, and on the other side, transplantation eliminates the need for hemodialysis, which might induce cognitive impairment because of hemodynamic changes [55,56,57,58,59]. Other studies have investigated patients with CKD undergoing peritoneal dialysis or hemodialysis or patients proposed for transplant whose cognitive performance was lower compared to patients without CKD [60]. During the dialysis process due to large changes and hemodynamic alterations that occur, there is a risk of increased acute cognitive impairment by the occurrence of acute cerebral ischemia [59]. Regarding the pathophysiology of cognitive decline in hemodialysis patients, systemic microvascular disease determined by inflammatory elements, hypertension, or diabetes, involving both the cerebral and renal area, could be a potential shared mechanism for the two conditions [61]. At the renal level, the degree of microvascular damage and the occurrence of microalbuminuria secondary to kidney injury may reveal comparable cerebral microvascular damage by disturbing the blood–brain barrier, the impairment of which causes protein leakage with cerebral white matter disease. This has been supported by brain imaging studies, which showed that progression of AD is enhanced in individuals with increased cerebro-spinal fluid/plasma albumin ratio [62,63].

Vascular stiffness well-defined as reduced vascular elasticity and prolongation of the duration of expansion of blood vessel as well as calcification expressed as significant deposition of calcium in the vascular wall are common traits of CVD [64]. Vascular stiffness is detected in the evolution of CKD and is substantially related with cognitive dysfunction [65]. Remarkably, vascular stiffness and impaired renal functions are strongly related to AD, whereby a vascular mechanism is largely implicated in the pathological processes of AD in CKD cases [66]. Moreover, a linkage between the renal function impairment and cognitive decline could be supported by the fact that the erythropoietin mainly synthetized at a renal level has neuroprotective effects, and its low levels reported in renal impairment, particularly in patients with renal insufficiency, may contribute to cognitive decline [67,68].

The endothelial interface, a synthetic bioreactor that generates various soluble factors, appears to be substantially functionally modified in neurodegenerative diseases, promoting a harmful central nervous system (CNS) environment by distributing neurotoxic and inflammatory species [69]. Markers of endothelial activation (EA) and dysfunction, such as plasma levels of von Willebrand factor (vWF), soluble vascular cell adhesion molecule-1 (sVCAM-1), soluble intercellular cell adhesion molecule-1 (sICAM-1), and sE-selectin were associated with reduced executive functioning and information processing speed in older subjects without [70] or with late-onset AD or vascular dementia [71]. The results of these studies, in addition to the vascular hypothesis, support the idea that elevated levels of EA markers are primarily implicated in the pathogenesis leading to cognitive decline [72].

### 2.2. Inflammation and Oxidative Stress

As the CNS immune system might be impaired by the inflammatory processes, the brain function can also be modulated by various mediators, such as pro-inflammatory cytokines, interleukines-1β (IL-1β), and the tumor necrosis factor (TNF), which may pass the blood–brain barrier (BBB) leading to neuropsychiatric alterations [40]. In patients with CKD, the concentrations of these cytokines, as well as elevated pro-inflammatory enzymes, such as inducible nitric oxide synthase (iNOS) and cyclooxygenase-2 (COX-2), appear to be positively controlled by the activation of nuclear-factor kappa-light-chain enhancer of activated B cells (NF-κB) [73]. The evidence of communication between the periphery and the CNS is the sickness behavior defined by behavioral changes, including neuropsychiatric developed in sick persons during inflammatory processes in the body [74]. Inappropriate and sustained activation of the innate immune system might be implicated in several neurologic diseases, among which is AD. The latest results of cytokine actions in the brain offer certain clues about the physiopathology of precise CNS disorders [75]. The potential mechanisms of kidney–brain crosstalk regarding inflammatory molecules are based on the fact that cytokines, such as IL-1β, interleukine-6 (IL-6), the TNF, and transforming growth factor β (TGF-β) frequently involved in the pathogenesis of CKD, may influence remote organs, such as the brain, reinforcing the idea of a kidney–brain inflammatory crosstalk [40,76].

**Figure 2:**
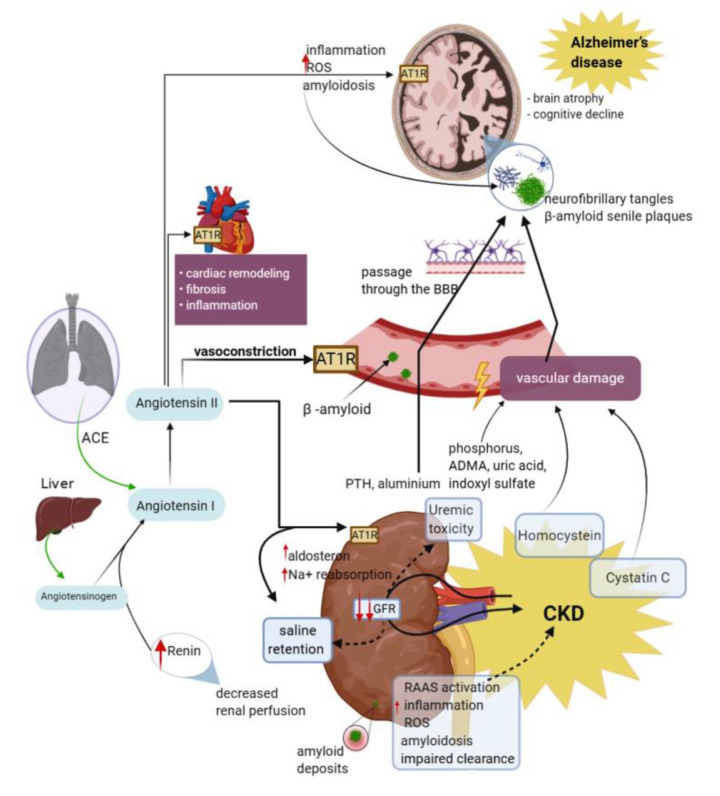
Schematic representation of mechanisms implicated in cognitive decline and AD in CKD patients, comprising renin–angiotensin system, oxidative stress, vascular injury, and inflammation. Diminished glomerular filtration rate and renal perfusion affect renal clearance. Renal retention of oxidizing substances promotes the intensification of oxidative stress and inflammation, which together with a defective clearance, causes an increase in plasma concentrations of uremic toxins, homocysteine, Cystatin C, amyloid deposits, and other molecules. These substances exert their toxic action through various mechanisms. Low renal perfusion leads to increased renin synthesis, and in its presence, the angiotensinogen synthesized in the liver is transformed into angiotensin I, which in turn, in the presence of angiotensin converting enzyme passes into angiotensin II. Angiotensin II activates AT1 receptors and produces hydro-saline retention, cardiac remodeling, vasoconstriction, and the development of β-amyloid deposits. The consequences of kidney damage are also reflected in the vascular and cerebral level with the aggravation of the cognitive deficit present in AD. The potential mechanisms of kidney–brain crosstalk regarding inflammatory molecules are because cytokines frequently involved in the pathogenesis of CKD may influence remote organs, such as the brain. The pathogenesis of the oxidative stress in patients with CKD is exhaustive, uremia and dialysis being among the important and frequent factors. Moreover, in these patients, an excessive increase in ROS was linked to the inflammatory processes, as the presence of endogenous oxidants and uremic toxins in the plasma may be a source of oxidative stress. Nevertheless, vascular damage and the direct neurotoxicity of uremic toxins produced by renal altered function are the most reasonable pathways of the effects of CKD in AD patients. AD, Alzheimer’s disease; ANG I, angiotensin I; ANG II, angiotensin II; ACE, angiotensin converting enzyme; RAAS, renin–angiotensin–aldosterone system; BBB, blood–brain barrier; GFR, glomerular filtration rate; AT1R, angiotensin II receptor type 1; ROS, reactive oxygen species; Na, sodium; ADMA, asymmetric dimethylarginine; CKD, chronic kidney disease.

Another important aspect related to the inflammatory theory is the oxidative stress defined to be the consequence of the imbalance between the oxidant system (production of free radicals) and antioxidant system, in favor of the oxidants, with destructive and pathogenic potential by disruption of proteins, lipids, and nucleic acids, with function losses and apoptosis [77], and other studies have shown that oxidative stress is related to the onset and development of diverse diseases like CKD and neurodegenerative disorders [40,78]. Various studies reported a reverse relationship between markers of oxidative stress and the filtration rate, which suggests that as the renal function is impaired, the species of free radicals gradually increase [78]. The pathogenesis of the oxidative stress in patients with kidney disorders is exhaustive, uremia and dialysis being among the important and frequent factors. As the renal function decreases, the antioxidant enzymes are modified, particularly in uremic patients. An increase in oxidative stress levels during hemodialysis might be due to the dialysis membrane and to the quantity of endotoxin during dialysis [54,74]. In CKD patients, excessive increase in reactive oxygen species (ROS) was linked to the inflammatory processes as the presence of endogenous oxidants and uremic toxins in the plasma may be a source of oxidative stress (Figure 2) [75]. In fact, reduction in nitric oxide bioavailability initiated by endothelial activation and dysfunction caused by oxidative stress stimulates the development of atherosclerosis. Increased ROS causes the inactivation and lack of nitric oxide, which is a key antioxidant in the protection of renal function by growing kidney blood flow, increasing natriuretic pressure, controlling tubule-glomerular activity, and maintaining electrolyte and fluid homeostasis. Deficiency of nitric oxide and elevated levels of plasma superoxide anion are suggested as essential promotors of oxidative stress [79]. A clinical study conducted by Vinothkumar et al. [80] determined the Aβ level in plasma in CKD and cognitive dysfunction patients. A total of 60 CKD patients, 30 CKD plus cognitive dysfunction patients and 30 control patients were included. The results of the study showed that enzymatic parameters, such as superoxide-dismutase (SOD), glutathione-peroxidase (GPX), catalase (CAT), and reduced glutathione (GSH), reported decreased levels in plasma and the lipoperoxidation level (LPX) being significantly increased in CKD plus cognitive dysfunction patients [80]. The LPX is a normal metabolic process in the life of a cell, but the excess is a pathogenic factor involved in more vascular and degenerative disorders. Counteracting the action of the oxygen-derived free radicals, which attack membrane phospholipids and initiate lipoperoxidation, both physiologically and pathologically, the body has a complex modulating and protective system, with an enzymatic component (SOD, GPX, GTP, CAT) and a non-enzymatic component (vitamin C, vitamin E etc.) [76]. In AD, an overexpression of the gene codifying SOD as well as in trisomy 21 was found. This enzyme is harmful due to the production of hydrogen peroxide, with the formation of superoxide ion. Based on the observations on Aβ peptide and on the role of free radicals in AD, Rose [81,82] showed that the formation of a Aβ peptide–ApoE complex might be favored by the presence of free radicals, and Buttefield et al. [83] showed that Aβ peptide in water solutions fragmentizes and generates free-radical peptides. This model could explain the slow onset of the disease: young people with higher antioxidant capabilities have a higher endurance to stress caused by free radicals, while aging associated with environmental stressors and genetic anomalies create favorable conditions for the onset of AD [74,83]. 

The evolution and severity of CKD are intensely related with the amplification of oxidative stress and inflammatory condition. These are recognized as risk factors for the onset of different systemic complications, such as cardiovascular disorders, mineral diseases, or anemia. Biological mechanisms comprising xanthine oxidase, mitochondrial activity, and nicotinamide adenine dinucleotide phosphate hydrogen oxidase appear to contribute to the onset and exacerbation of oxidative stress and inflammation [84].

### 2.3. The Renin–Angiotensin–Aldosterone System (RAAS)

The renin–angiotensin–aldosterone system (RAAS) is an endocrine system widely known for its physiological roles in electrolyte homeostasis, body fluid volume regulation, and cardiovascular control [85], with renin and angiotensin being critical factors [86]. Renin, an enzyme produced by the kidney, acts on angiotensinogen (AGT), a liver-precursor, transforming it into angiotensin I (ANGI), a decapeptide. The levels in plasma of the angiotensinogen may be increased by high levels of corticosteroids and other hormones. Another enzyme, angiotensin converting enzyme (ACE), cleaves ANG I to the active octapeptide ANG II, predominant at lung, heart, cardiac, renal, adrenal, and brain level, with a variety of functions, such as vasoactive on all blood vessels; stimulation of adrenal glands with aldosterone release involved in maintaining sodium–potassium homeostasis by stimulation of proximal tubules; stimulation of antidiuretic hormone (ADH), as well as playing a role in the cognitive processes (memory and learning) (Figure 2) [85,86,87,88]. ANG II may influence the release of prostaglandins with the occurrence of renal vasoconstriction. ANG II degradation with ACE enzyme is the basis of treating diabetic nephropathy, and its deficit has been associated with albuminuria and glomerular injury. Use of angiotensin converting enzyme inhibitors and angiotensin II receptor blockers together with a low sodium intake proved to have beneficial effects on renal pathology [87].

RAAS activation also mediates brain level effects, such as neuronal damages, as this system is expressed in the CNS. ANG II, the key element of the system also mediates the progression of AD [86]. Discovery of the RAAS independent of the peripheral system encouraged several investigators to focus on this system with implications in the brain function and disorders and in neuroprotection but with possible roles in the etiology of the AD as well. An important factor in AD pathology is the chronic stress facilitating the increase in brain ANG II level; a tight relationship between ANG II increased levels and amyloidgenesis has been suggested. This hypothesis has been consolidated by various recent studies that reported the beneficial effects on the cognitive processes or even the diminishing of Aβ oligomerization on AD animal models, following treatment with angiotensin II receptor blockers [85,89]. The chronic activation of RAAS followed by the increase in ANG II level and activation of angiotensin II receptor type 1 may lead to the occurrence of various physiopathological processes, such as vasoconstriction, inflammation, high sodium renal intake, and fibrosis. Similarly, it has been found that all the components of the brain renin–angiotensin system may be synthesized locally in the brain. More studies reported that both renin and angiotensinogen have been detected in brain cells, stimulating renin signaling and determining cognitive impairment by activation of angiotensin receptors [90]. RAAS receptors have been observed in several brain areas, including the hippocampus and the cingulate cortex [91]. In the brain, angiotensin II type 2 receptor activation contributes to the control of the cerebral circulation, central sympathetic activity, integrity of the BBB, and the regulation of the brain’s innate immune response [92]. Brain over-activation of angiotensin II receptors is associated with pathological processes, including inflammation and cognitive decline, as it occurs in AD [93]. The brain renin–angiotensin system might be a risk factor for oxidative stress, which mediates the brain function as increased ANG II stimulates superoxide generation by inflamed cells causing death of dopaminergic neurons [85]. 

The relationship between cognitive decline and impaired kidney function appears to be bidirectional. Understanding the pathology of the relations between these two conditions is essential to preclude and/or reduce the incidence and influence of cognitive damage in CKD patients. 

## 3. The Relationship between Biomarkers Related to Alzheimer’s Disease and Renal Function

### 3.1. The Potential Pathophysiological Markers Associated to Alzheimer’s Disease

Pathologically, AD is characterized by the accumulation of Aβ peptides, visualized as senile plaques and hyper-phosphorylated Tau proteins, which appear as neurofibrillary tangles located in the neocortex and the hippocampus [2]. The intracellular neurofibrillary tangles are composed of paired helical filaments formed of hyper-phosphorylated Tau protein aggregates, and the senile plaques are rich in Aβ, which can be secreted by neurons directly into the cerebro-spinal fluid (CSF) [94]. Aβ is a peptide formed of 40–42 amino acids, with a molecular weight of approximatively 4 kDa, that is derived from the proteolytic cleavage of APP by the action of β and γ-secretases [83,95]. It is worth mentioning that, elevated serum Aβ levels were also recorded in CKD individuals, probably due to the reduced clearance of Aβ protein overproduction in the blood of these patients, leading to the idea that cognitive damage and AD related to impaired renal function may be influenced by Aβ protein in people with CKD [96]. Moreover, in recent years, more studies support that the CSF Aβ1-40/Aβ1-42 levels are better predictors of AD progression than plasma Aβ isoforms [94,97]. Clinico-pathological correlation studies have revealed that a growth in tau protein/phosphorylated tau in the CSF represents a sign of AD and correlates with the degree of dementia [98,99]. Furthermore, studies revealed that changes in the Aβ deposition and tau protein can be linked to the AD pathology after the increase in aluminum in dialysis patients [100]. 

Homocysteine (Hcy), a key molecule formed during the metabolism of sulfur-containing amino acids, is a direct and modifiable risk factor of early cognitive impairment, being involved in AD pathogenesis [101,102] through the dysfunction of endothelial cells and small blood vessels by oxidative stress [4,103]. Hyperhomocysteinemia, a condition of high levels of Hcy in the blood, is a frequent clinical finding in patients with CKD or acute kidney injury (AKI), probably due to impaired Hcy clearance in individuals with renal impairment [104,105]. The increase in Hcy levels determined by CKD has been correlated to the decrease in Aβ1-42 in the CSF, suggesting that serum Hcy levels may be a potential marker of AD in CKD patients [97]. 

Recent advances in blood biomarkers of cognitive dysfunction have demonstrated that Glycogen synthase kinase-3β (GSK3β), a serine/threonine kinase, plays a notable role in the AD pathogenesis. Excessive activation of GSK3β is accompanied by Aβ production and hyper phosphorylated tau [106]. The relationship between GSK-3β, total tau, p-Tau 181 levels, Aβ and neurodegeneration, investigated mostly in cases with mild cognitive dysfunction [107,108] or mild AD, reflects a robust negative correlation between abnormal proteins concentrations with the Mini Mental State Exam and Wechsler Memory Scale-I and a positive one with Tower of London test [109]. Detection and use of uncharacteristic protein levels, preferable in relation to neuropsychological screening, appear to be a possible instrument that may improve the CKD in connection with cognitive decline diagnosis. Several other blood biomarkers of AD cognitive decline have been suggested. Increased plasma neurofilament light chain (NfL) concentrations are highly correlated with cerebrospinal fluid levels [110], which might be comparable in relation to diagnostic efficiency with Aβ1-42:Aβ1-40 plasma ratio [111]. Stevenson et al. [112] pointed out that the intrinsic pathophysiological features of dementia, including AD, can be reflected by erythrocytes. Furthermore, the authors also suggested that the morphology of erythrocyte and their protein levels, such as calpain-1, Hsp90, and IgG A, were relevant blood biomarkers for AD. Plasma evaluation of over 50 categories of inflammatory proteins in individuals with cognitive decline and compared them with healthy controls revealed that FH (factor H) and FB (factor B) could predict the evolution of mild cognitive impairment to AD [113]. However, the specificity of these blood biomarkers of AD cognitive dysfunction has not been rigorously studied, and they remain frequently in the discovery phase of development. Elevated plasma levels of α1-antichymotrypsin have been validated in AD with fluctuating degrees of correlation to disease progression [114], but this result was not sustained by a third study [115].

### 3.2. Potential Use in the Diagnosis of Alzheimer’s Disease of the Biomarkers Associated with Chronic Kidney Disease 

A review of the existing data regarding uremic toxicity identified more than 90 different types of urinary toxins in CKD individuals, however their impact on the body remains unclear [116]. Low kidney perfusion and severe GFR reduction are the most frequent reasons for increased and accumulated uremic toxins in CKD cases. Lately, increased attention has been focused on small and intermediate-sized molecule toxins, such as phosphorus, parathyroid hormone (PTH), asymmetric dimethylarginine (ADMA), uric acid, indoxyl sulfate, and aluminum [117,118]. Recent studies have shown a substantial elevation in PTH levels in CKD patients’ blood, and this hormone is closely associated with a cognitive decline and AD [119,120,121,122]. Given the ability of PTH to cross the BBB and the receptors for this hormone being disseminated in the brain, a parathyroidectomy performed in CKD patients with secondary hyperparathyroidism may attenuate the cognitive impairment [123]. The mechanisms proposed to elucidate the possible links between PTH, cognitive damage, and AD include the role of PTH in regulation of intra- and extracellular calcium and decrease local brain blood flow by increased the concentration levels of PTH [123,124,125]. In addition, ADMA and hyperphosphatemia have been confirmed to be involved in the development of renal failure and vascular injury [126,127,128]. Hyperuricemia, a mediator or an independent risk factor for the development and progression of renal disease, is closely associated with brain atrophy and memory decline in CKD [129,130]. Pathophysiological mechanisms underlying these effects initiated by uric acid are an activation of RAAS, augmented oxidative stress, endothelial dysfunction, proinflammatory, and proliferative actions [131,132]. In CKD, the reduced capacity to remove protein-binding agents, such as indoxyl sulfate, can promote inflammatory genes expression and oxidative stress, leading to cognitive dysfunction [133,134]. Moreover, indoxyl sulfate causes inflammation, amplifying the interaction between endothelial cells and leukocyte, which is notable in the AD development [135]. Elevated brain deposits of aluminum caused dementia in dialysis patients [54] and are correlated with high mortality rates. The complications described in response to exposure to aluminum-containing phosphate binders or to water used in dialysis preparations containing high levels of aluminum could be eliminated by strict water tests and limiting the use of aluminum-based phosphorus binders [136,137].

Cystatin C, another marker of kidney function appears to be less influenced by muscle mass, being an important predictor of clinical results linked to AKI and CKD than creatinine, although its clinical role is not yet clearly defined [138,139]. The co-localization of cystatin C, a protein encoded by the CST3 gene, a sensitive gene of late-onset AD with Aβ in parenchymal and vascular amyloid deposits in the brains of individuals with AD, may reveal cystatin C contribution in amyloidogenesis [140,141]. Recent studies have highlighted that cystatin C shows a controversial influence in the pathology of AD; on the one hand, it appears to control the levels of Aβ that bind directly to Aβ and inhibit its aggregation, but on the other hand, as a substrate for cathepsin B protease, it appears to be competitive for Aβ degradation [142,143,144]. To date, it is not known whether cystatin C brain deposition leads to a reduction in cystatin C in peripheral plasma or whether in patients with CKD increased plasma cystatin C concentrations would stimulate precipitation and binding of cystatin C and APP in the brain. Serum cystatin C levels may be a novel possible biomarker of AD in CKD people. Additionally, proteinuria and eGFR are recognized as common indicators for renal function [145,146] are closely associated to a cognitive decline [25,36]. 

## 4. Effects of Renal Replacement Therapies on Amyloid-Beta Pathology

As AD still remains irreversible, studies continue to explore modifiable risk factors as a promising safe approach for AD prevention and therapy, which could lead to the avoidance of side effects related with the entry of Aβ-targeted compounds into the brain. To date, restoring the function of Aβ clearance is considered a valuable strategy for treating AD [147]. It has been shown that elevated Aβ production or deficits in Aβ clearance play key or causal roles in the pathogenesis of AD. In patients diagnosed with AD, Aβ clearance via the BBB was estimated to be decreased by approximately 30% [148]. Despite previous studies on murine and human BBB models that have highlighted that about 40–60% of brain-derived Aβ is cleared in the periphery, the amount and mechanisms are still poorly understood [149,150]. Deep cervical lymph node ligation intensified AD-like pathology in APP/PS1 mice exhibiting more severe cerebral Aβ deposition, synaptic protein loss, neuroinflammation, decreased polarization of aquaporin-4, and exploratory and cognitive behaviors deficits [151]. Jin et al. [152] in a clearance of brain-derived Aβ study found that peritoneal dialysis, a clinically existing therapeutic method for CKD, significantly reduced Aβ deposition, also attenuating other AD-type pathologies, such as neuroinflammation, tau hyperphosphorylation, glial activation, synaptic dysfunction, and neuronal loss and attenuated the behavioral impairment in APPswe/PS1 mice.

Liu et al. [153], in a study on the potential implications of renal roles in Aβ clearance, revealed that serum Aβ1-40 and Aβ1-42 levels measured were significantly higher in CKD patients (31 dialyses cases and 16 non-dialyses individuals) than in healthy control subjects. Moreover, CKD individuals receiving peritoneal dialysis registered inferior levels of Aβ than non-dialysis cases, being comparable to those of healthy controls and have been correlated with renal function as evaluated by eGFR and residual GFR. The cause of these higher concentrations of Aβ in patients with renal dysfunction without dialysis is not fully known. These results sustain the main role of kidney in Aβ hemostasis and the idea that impaired renal function could lead to inadequate Aβ clearance and thus contribute to the AD development [154,155].

A prospective study on plasma Aβ levels and cognitive function on CKD patients performed during the period between baseline and long-time hemodialysis (18 or 36 months) showed that Aβ1-40 levels diminished or did not change, while Aβ1-42 levels remained unchanged or tended to increase significantly at the second time point. In most patients without broad cerebral white matter modifications, Mini Mental State Examination scores elevated or were maintained at 18 months follow-up. These findings suggest that cognitive decline as a consequence of cerebrovascular disease has not been improved by hemodialysis [156]. A rapid decrease in the blood of Aβ concentrations can act as a trigger to increase Aβ excretion from the brain, leading to attenuation of the cognitive decline. Thus, previous studies using a kinetic analysis described that hemodialyzers were able to reduce Aβ1-42 and Aβ1-40 by 32.7% and 51.1%, respectively, inducing an extensive influx of Aβ into the blood during hemodialysis periods [157,158]. In contrast, elevated blood Aβ levels and impaired cognitive performance along with decreased renal function in CKD patients without hemodialysis [158].

In a similar study by Tholen et al. [159], assessing cognition and plasma Aβ concentrations in cognitively impaired hemodialysis patients, the total clearance rates of plasma Aβ1-40 and Aβ1-42 were about 35% and 28%, respectively, with a significant reduction in the first 2 hours of hemodialysis. Moreover, Aβ1-42, not Aβ1-40, baseline levels were significantly correlated with cognitive function using the Montreal Cognitive Assessment. In line with these results, renal clearance of Aβ could be vital in maintaining cognitive performance and peripheral Aβ decrease by hemodialysis and, in the future, could serve as an anti-amyloid therapy strategy, despite its usefulness in AD patients.

## 5. Conclusions

The pathophysiology and neuropathological substrates shared by brain and kidney damage are robust and complex. The cognitive decline and AD registered in CKD patients may be explained by the susceptibility of brain tissue to vascular dysfunction, inflammation, oxidative stress, and the renin–angiotensin–aldosterone system. Prompt recognition and management of these mechanisms in first CKD stages may signify a window of opportunity to diminish their influence at advanced stages. Increasingly detailed exploration of the molecular relationship between renal failure and brain function, as a shared pathological feature in both CKD and AD, is crucial in order to diminish the risk for future cognitive deficits and may provide new directions for development of therapies and biomarkers that can preclude or diminish the occurrence of CKD, as well as AD. Additionally, the setting of renal impairment could be evaluated as a potential determinant for cognitive damage in Alzheimer’s disease. We could acquire surprising findings if we center our attention on alterations that occur in both the kidneys and the brain in additional research in the prevention and therapy of AD and CKD.

## Figures and Tables

**Table 1:** Chronic kidney disease staging definitions and correspondence with Kidney Disease Outcomes Quality Initiative (KDOQI) Clinical Practice Guidelines categories.

Chronic Kidney Disease Stages	Description	Potential Sign/Symptoms
Stages 1 and 2	minimal kidney damage with normal eGFR >60 mL/min/1.73 m^2^, ACR ≥30 mg/g	usually urea and creatinine levels are normal or slightly raised
Stage 3	moderate reduced eGFR 30–59 mL/min/1.73 m^2^	early signs occur and may comprise fatigue and weakness, loss of appetite, itching, rising levels of urea and creatinine, anemia, nausea, vomiting, hypertension
Stage 4	severe reduced eGFR 15–29 mL/min/1.73 m^2^	anemia, hypertension, nausea, vomiting, reduction in calcium absorption, dyslipidemia, heart failure, metabolic acidosis
Stage 5	kidney failure, eGFR <15 mL/min/1.73 m^2^	anemia, hypertension, nausea, vomiting hypertrophy of left ventricular, hyperparathyroidism, hyperphosphatemia, hyperkalemia
End-stage renal disease (ESRD)	renal transplant and dialysis	Anemia, cardiovascular dysfunction, hyperparathyroidism, hyperphosphatemia, hyperkalemia

eGFR estimated glomerular filtration rate; ESRD, end-stage renal disease, ACR, albumin: creatinine ratio.

## Abbreviations

AD	Alzheimer’s disease
APP	amyloid precursor proteins
CKD	chronic kidney disease
ApoE4	epsilon 4 allele of the apolipoprotein E gene
SorLA	sorting protein-related receptor
eGFR	estimated glomerular filtration rate
ESRD	end-stage renal disease
CVD	cerebrovascular disease
IL-1β	interleukines-1β
IL-6	interleukine-6
TNF	tumor necrosis factor
BBB	blood brain barrier
CNS	central nervous system
TGF-β	transforming growth factor β
ROS	reactive oxygen species
SOD	superoxide-dismutase
GPX	glutathione-peroxidase
CAT	catalase
GSH	glutathione
LPX	lipoperoxidation level
RAAS	renin–angiotensin–aldosterone system
AGT	angiotensinogen
ANG I, II	angiotensin I, II
Aβ	beta-amyloid
ACE	angiotensin converting enzyme
ADH	antidiuretic hormone
Hcy	homocysteine
PTH	parathyroid hormone
ADMA	asymmetric dimethylarginine
RAAS	renin–angiotensin–aldosterone system
vWF	von Willebrand factor
sVCAM-1	soluble vascular cell adhesion molecule-1
sICAM-1	soluble intercellular cell adhesion molecule-1
EA	endothelial activation
GSK3β	glycogen synthase kinase-3β
NfL	neurofilament light chain
FH	factor H
FB	factor B
iNOS	inducible nitric oxide synthase
COX-2	cyclooxygenase-2
NF-κB	nuclear factor kappa-light-chain-enhancer of activated B cells.
